# Experimental and Theoretical Studies on Reaction Kinetics, Mechanism, and Degradation of Quinoline‐Based Herbicide with Hydroxyl Radical, Sulphate Radical Anion, and Hydrated Electron

**DOI:** 10.1002/cphc.202401135

**Published:** 2025-05-01

**Authors:** Beena G. Singh, Hari P. Upadhyaya

**Affiliations:** ^1^ Radiation & Photochemistry Division Bhabha Atomic Research Centre HBNI Trombay Mumbai 400 085 India

**Keywords:** density functional calculations, oxidative degradation pathways, pulse radiolysis, quinoline‐based herbicides, reaction mechanisms

## Abstract

The kinetic and mechanistic studies for the reaction of hydroxyl radical (^•^OH), sulfate radical anion (${\text{SO}}_{4}^{•-}$), and hydrated electron $\left(e_{\text{aq}}^{-}\right)$ with quinoline‐based herbicide, namely, 8–quinoline carboxylic acid (8QCA), have been performed using experimental and computational methods. Experimental studies are performed using pulse radiolysis technique at different pHs and corroborated with theoretical studies using ab initio molecular orbital calculations. At lower pH of 1, the 8QCA is protonated and reacts with ^•^OH radical to generate transient spectrum with maxima at 340 and 420 nm. Similarly at higher pH of 9, the 8QCA is deprotonated and shows transient absorption maxima at 320 nm. At neutral pH, it exists as neutral species and reacts with ^•^OH radical differently. Theoretically, individual rate coefficients for ^•^OH radical addition reaction with each carbon atoms are evaluated including solvent effect and tunneling correction. Fukui index and individual rate constant determination confirm that C5 carbon atom is the most reactive site for the ^•^OH radical addition reaction. The total rate constant evaluated theoretically and experimentally for the ^•^OH radical reaction is equal to its diffusion‐limit value. The ability of ^•^OH radical to degrade 8QCA is found to be higher as compared to $e_{\text{aq}}^{-}$.

## Graphical abstract

Advanced oxidative process is commonly used for the degradation/removal of herbicides from soil/water bodies. It mostly involves oxidizing ^•^OH and ${\text{SO}}_{4}^{•-}$ species. The present study involves an experimental and theoretical approach to explore kinetics, mechanism, and degradation pathways for 8–quinoline carboxylic acid (8QCA), which has basic structure for two herbicides, namely, quinclorac and quinmerac.

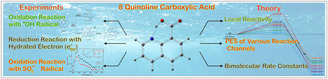

## 1 Introduction

In the modern world, the usage of herbicide is widespread starting from crop farming, lawns, parks to golf courses, and other areas.^[^ ^1^, ^2^, ^3^ ^]^ Quinoline‐based herbicides can be considered as one of the widely consumed herbicides which come under the category of auxin mimics.^[^ ^4^, ^5^ ^]^ All over the world, the major usage of these quinoline‐based herbicides is in the agricultural productions which not only improves the quality but also the quantity of the crops. However, the excessive application of these herbicides poses enormous potential risks ranging from contamination of groundwater, loss of soil fertility, nitrate leaching, and finally the loss of biodiversity.^[^ ^6^, ^7^ ^]^ These herbicides are also chemically stable molecules which poses considerable risks to the ecosystem and human health.^[^ ^8^ ^]^ Hence, it is desirable to study their environmental impacts and different effective removal strategies.^[^ ^9^ ^]^ In this context, the present study revolves around the oxidation/reduction and degradation process of 8–quinoline carboxylic acid (8QCA) which has a basic structure of two very important herbicides, namely, quinclorac (3,7‐dichloro 8–quinoline carboxylic acid) and quinmerac (7–chloro–3–methyl 8–quinoline carboxylic acid).

Due to their low p*K*_a_ values, the quinoline acid‐based herbicides get deprotonated forming anionic species and become extremely soluble in water which increases their mobility.^[^ ^10^ ^]^ This augments their availability for its degradation processes. Various types of degradation processes are applied for these herbicides which are mainly based on biological degradation methods,^[^ ^11^, ^12^, ^13^, ^14^, ^15^, ^16^, ^17^ ^]^ chemical methods,^[^ ^18^, ^19^ ^]^ and photodecomposition methods.^[^ ^20^, ^21^, ^22^, ^23^, ^24^ ^]^ In many instances, it has been observed that a combination of chemical method with photocatalysis is also used widely and considered as the most effective oxidative degradation process.^[^ ^25^ ^]^ This is often known as advanced oxidation process (AOP).^[^ ^26^, ^27^, ^28^, ^29^ ^]^ The AOP involves the generation of highly reactive oxidizing species, such as hydroxyl radical (^•^OH) and sulphate radical anion (${\text{SO}}_{4}^{•-}$) which finally degrades these organic contaminants. Hence, it is desirable to know complete knowledge of the initial mode of attack, the rate constant, and the nature of final product in the reaction of various oxidants with these herbicides. This type of study will lead us to understand their degradation mechanism and their lifetime in the environment.

In the literature, one can find few studies on degradation process of 8QCA‐based herbicides, namely, quinclorac and quinmerac.^[^ ^24^, ^30^, ^31^, ^32^ ^]^ Utilizing a biotransformation strategy with Burkholderiacepacia WZ1, Li et al. studied its biodegradation process.^[^ ^33^ ^]^ Similarly, Rohers et al. studied the bioremediation of quinclorac in water using a combination of aquatic plants and bacteria.^[^ ^34^ ^]^ Few other studies are also available in the literature in the area of bioremediation of quinclorac using various bacteria and bionanocomposite.^[^ ^31^, ^35^ ^]^ Similarly Pareja et al. studied the photolytic and photocatalytic degradation of quinclorac in ultrapure and paddy field water using AOP.^[^ ^24^ ^]^ In one study, carbon‐coated nitrogen‐doped TiO_2_ was used for the photocatalytic degradation.^[^ ^30^, ^36^ ^]^ Furthermore, permanganate and nano‐Fe^0^/peroxymonsulfate system have been used in some of the degradation studies.^[^ ^32^ ^]^ A few studies also involved using the cobalt (0/II) incorporated N‐doped porous carbon and gold nanocatalyst for the degradation process of quinclorac.^[^ ^36^, ^37^ ^]^ In the theoretical side, chemical reactivity of quinclorac was studied employing the hard–soft acid–base (HSAB) local principle by computing Fukui function.^[^ ^38^ ^]^ Apart from this lone theoretical study, the literature is devoid of any such theoretical study on the degradation of quinclorac. Similar to quinclorac, experimental study available in the literature is by Despotović et al. where they have studied the photocatalytic degradation of quinmerac in various types of natural water utilizing TiO_2_ suspensions.^[^ ^39^ ^]^ On the theoretical side, very recently Ngo et al. studied the oxidation process of ^•^OH radical with quinmerac in aqueous phase.^[^ ^40^ ^]^ In our recent theoretical study, we have studied kinetics, mechanism, and degradation pathways of quinclorac and quinmerac herbicides with the ^•^OH radical reaction in aqueous media.^[^ ^41^ ^]^ In this context, the present study is carried out to understand the kinetics, mechanism, and the degradation pathways for 8QCA with ^•^OH radical, sulphate radical anion (${\text{SO}}_{4}^{•-}$), and $e_{\text{aq}}^{-}$ using experimental methods. Theoretically, the present endeavor is a sincere effort to throw some light on the reaction mechanism, specially the initial mode of attack of ^•^OH radical, the formation of ^•^OH radical complex, and the ^•^OH radical total rate constant in liquid phase. Moreover, branching ratio has been also calculated theoretically and discussed in light of the local reactivity parameters calculated using the concept of Fukui indices.

## 2 Results and Discussion

### 2.1 Experimental

**Scheme 1:**
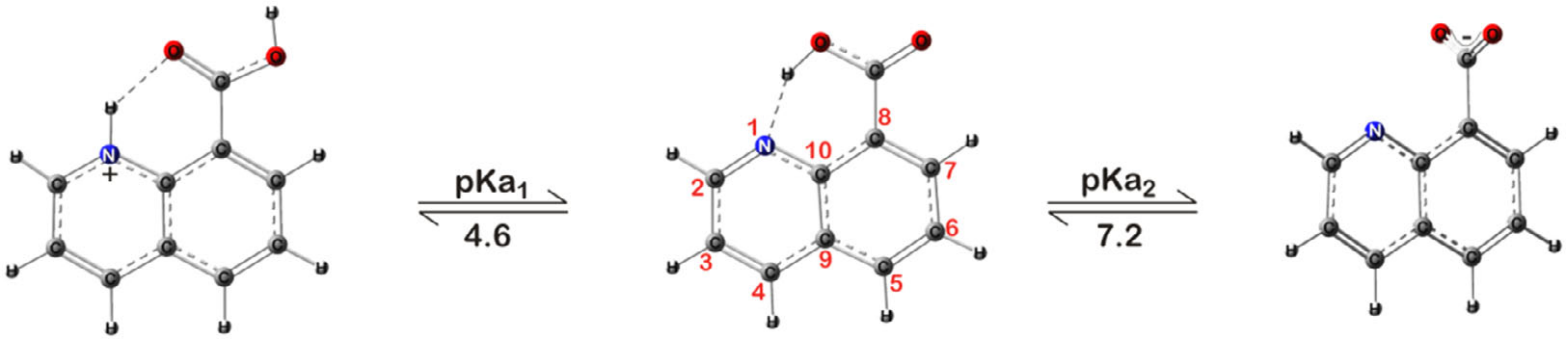
Structure of 8QCA with ring atom numbering and its pH‐dependent transformation.

The absorption spectrum of 8QCA exhibits pH‐dependent spectrum showing absorbance in the wavelength range of 200–400 nm with absorption maxima at 207, 235, and 315 nm at pH 7 and the absorption maxima at 207, 230, and 290 nm at alkaline pH (≈9). The pKa of 8QCA is reported to be at 7.2 and 4.6, which is attributed to the carboxylic group and the nitrogen of the quinoline ring, respectively. The prototropic equilibrium in which 8QCA exists is given in **Scheme** **1**.

At pH above 7.2, 8QCA has a single negative charge whereas at pH below 2.6, it is in positively charged. These different forms are expected to show pH‐dependent reaction with ^•^OH radical. Hence, ^•^OH radical reactions were performed in the pH range from 1 to 10 to further explore its detailed mechanism. The detailed observation is discussed in the following paragraphs.

### 2.2 Reactions of ^•^OH Radical with 8QCA

**Figure 1:**
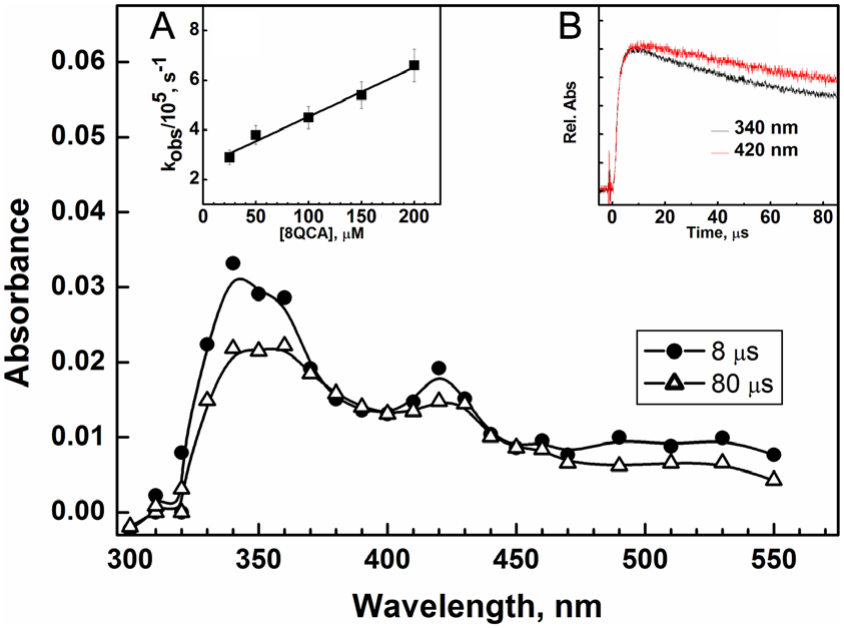
Transient absorption spectrum obtained on pulse irradiating N_2_ saturated aqueous solution containing 0.1 mM 8QCA at pH 1. Inset (A) shows the observed rate at 340 nm against 8QCA concentration. Inset (B) shows the time evolution of absorption at 340 nm (red line) and 420 nm (black line).

At pH 1, 8QCA will have a single positive charge with both the carboxylate and the nitrogen in the protonated state. The reaction of ^•^OH radical at pH 1 was studied by pulse radiolyzing N_2_ saturated aqueous solution containing 8QCA (0.1 mM). As seen in **Figure** **1**, the resulting transient absorption spectrum exhibited a broad absorbance in the range from 300 to 550 nm with maxima at 340 and 420 nm. The observed rate, estimated by following the buildup kinetics at 340 nm, exhibited a linear relationship with the concentration of 8QCA, yielding a bimolecular rate constant of 1.9 × 10^9^ M^−1^ s^−1^ (inset A of Figure 1). This indicates that the reaction between ^•^OH radicals and the substrate at pH 1 occurs via a diffusion‐controlled process. The absorbance at 340 and 420 nm decayed by following first‐order kinetics with rate constant of 1.0 × 10^4^ and 0.89 × 10^4^ s^−1^. Using the decay constant, the half‐life of the transient at 340 and 420 nm were calculated to be ≈70 and 78 μs, respectively. From the difference in the decay rate constant of ≈20%, it can be deduced that both the absorbance maxima may arise due to different transients. Additionally, the overlay of the relative absorbencies at these wavelengths also confirms that the nature of the transient is different (inset B of Figure 1).

**Figure 2:**
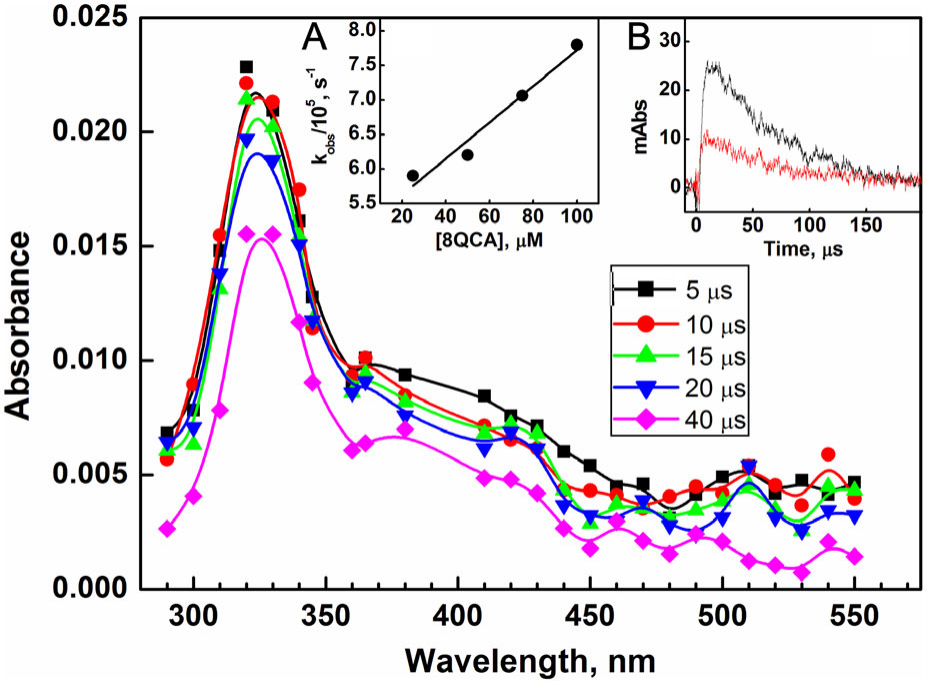
Transient absorption spectrum obtained on pulse irradiating N_2_O saturated aqueous solution containing 0.1 m 8QCA at pH 10. Inset (A) shows the observed rate at 320 nm against 8QCA concentration. Inset (B) shows the absorption‐time plot at 320 nm (black line) and 420 nm (red line).

Since the second p*K*_a_ of 8QCA is 7.2, the singly deprotonated form (anionic form) of 8QCA will predominate at pH values above 9.2. Therefore the reaction of 8QCA with ^•^OH radical was studied at pH 10. For this, the transient spectrum was generated by pulse radiolyzing N_2_O saturated aqueous solution containing 8QCA (0.1 mM) at pH 10. As seen in **Figure** **2**, the transient absorption spectrum recorded at 5 μs after the pulse exhibited absorption maximum (*λ*_max_) at 320 nm with an additional broad featureless absorption ranging from 370 nm to 470 nm. The well‐defined band seen at 420 nm at pH 1 is not clearly visible at alkaline pH. The formation rate constant at pH 10 (k_2_) of this transient was determined from the slopes of the linear plots of observed pseudo‐first‐order build‐up rate constants at 320 nm against 8QCA concentration (inset in A of Figure 2). The k_2_ was determined to be (2.6 ± 0.2) × 10^9^ M^−1 ^s^−1^, which is comparable to that observed at pH 1. The difference in the spectrum accompanied by the change in the rate constant for the ^•^OH radical scavenging confirms that the reaction of ^•^OH radical with 8QCA is pH dependent. Further, the transient absorbing at 340 nm followed first‐order decay kinetics with a rate constant of (1.7 ± 0.1) × 10^4^ s^−1^, using which, the half‐life of transient was calculated as 40 μs (Inset B of Figure 2).

**Figure 3:**
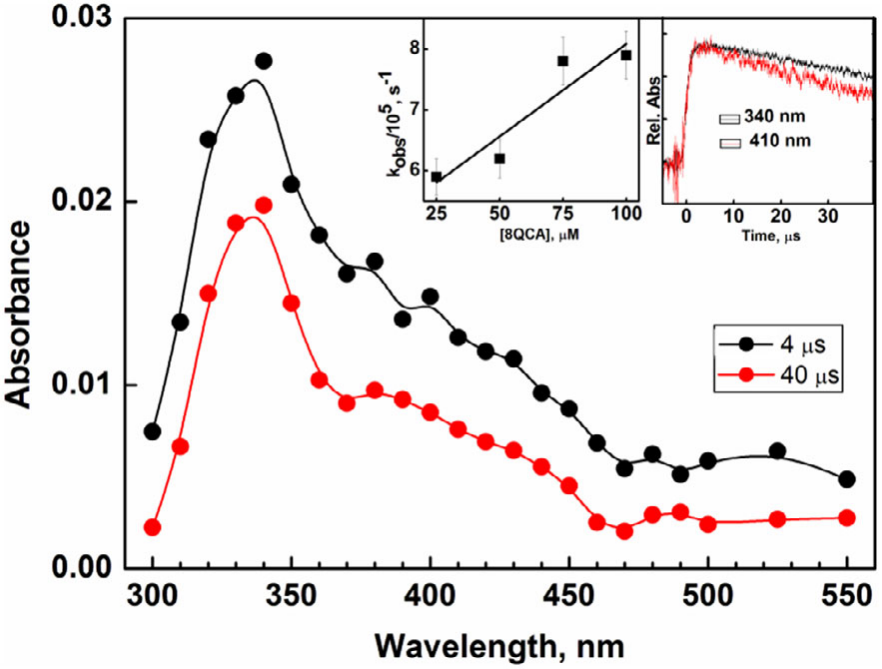
Transient absorption spectrum obtained on pulse irradiating N_2_O saturated aqueous solution containing 0.1 mM 8QCA at pH 7 (buffered with phosphate) at different time delays.

Similarly, the reaction of 8QCA with ^•^OH radical was also studied at a neutral pH of 7. At neutral pH, the 8QCA exists as neutral form. As seen in **Figure** **3**, the transient absorption spectrum recorded at 5 μs after the pulse exhibited absorption maximum (*λ*_max_) at 320 nm with an additional broad featureless absorption ranging from 370 to 450 nm. The formation rate constant at pH 7 of this transient was determined from the slopes of the linear plots of observed pseudo‐first‐order build‐up rate constants at 320 nm against 8QCA concentration. The bimolecular rate constant so obtained at pH 7 is determined to be (6.2 ± 0.4) × 10^9^ M^−1 ^s^−1^, which is higher than that observed at pH 1 and pH 10.

Additionally, the spectrum of ^•^OH radical with 8QCA was also recorded at pH 4.5 and 8.7 (Figure S1, S2, Supporting Information). The spectrum generated at pH 4.5 and pH 7, showed absorption maximum at 340 nm, while that at pH 8.7 exhibits a peak at 330 nm. The absorption band in the range 380–450 nm became broad and featureless with increase in pH. In general, radical cations are stabilized by the presence of electron‐donating groups near the charged center or under acidic conditions. In the case of 8QCA, the carboxylic group at the 8‐position shifts the natural pKa of the carboxyl group from 4 to ≈7. This electron‐withdrawing carboxyl group destabilizes the radical cation in 8QCA, leading to its hydrolysis and formation of OH‐adducts. As a result, the absorbance peak at 420 nm, which is most prominent at acidic pH and diminishes as pH increases, can be attributed to the radical cation of 8QCA.^[^ ^42^, ^43^ ^]^ The rate constant for the reaction was found to increase with increase in pH till 8.7 (1.7 ± 0.2 × 10^10^ M^−1 ^s^−1^), while at pH 10 the rate constant was slightly lower (2.7 ± 0.3 × 10^9^ M^−1 ^s^−1^). This may be due to the contribution of O^•−^ radical at the alkaline pH. Similarly, the half‐life of the transient in the UV region (320–340 nm) decreased with increase in pH.

**Figure 4:**
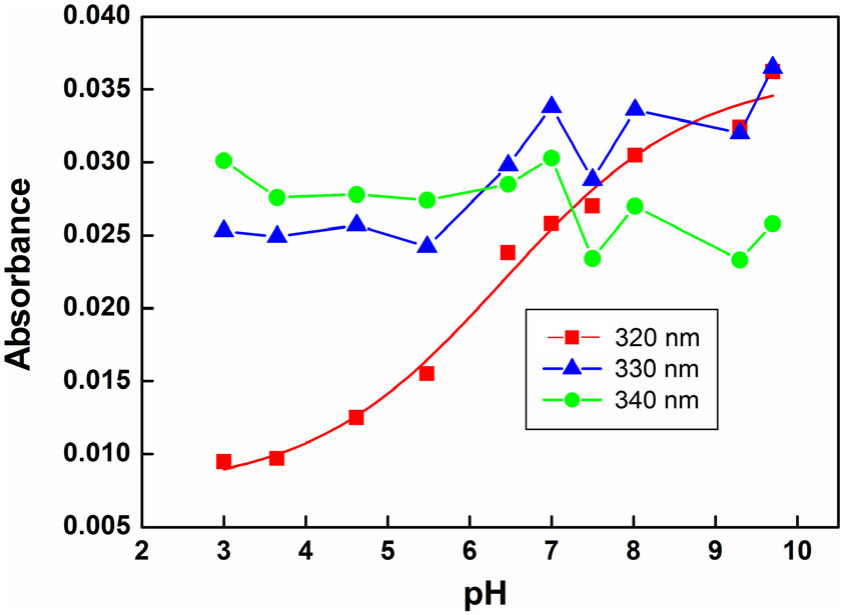
Plot of pH‐dependent absorbance at 320, 330, and 340 nm, corresponding to the transient species formed during the reaction of 8QCA with ^•^OH radicals.

**Scheme 2:**
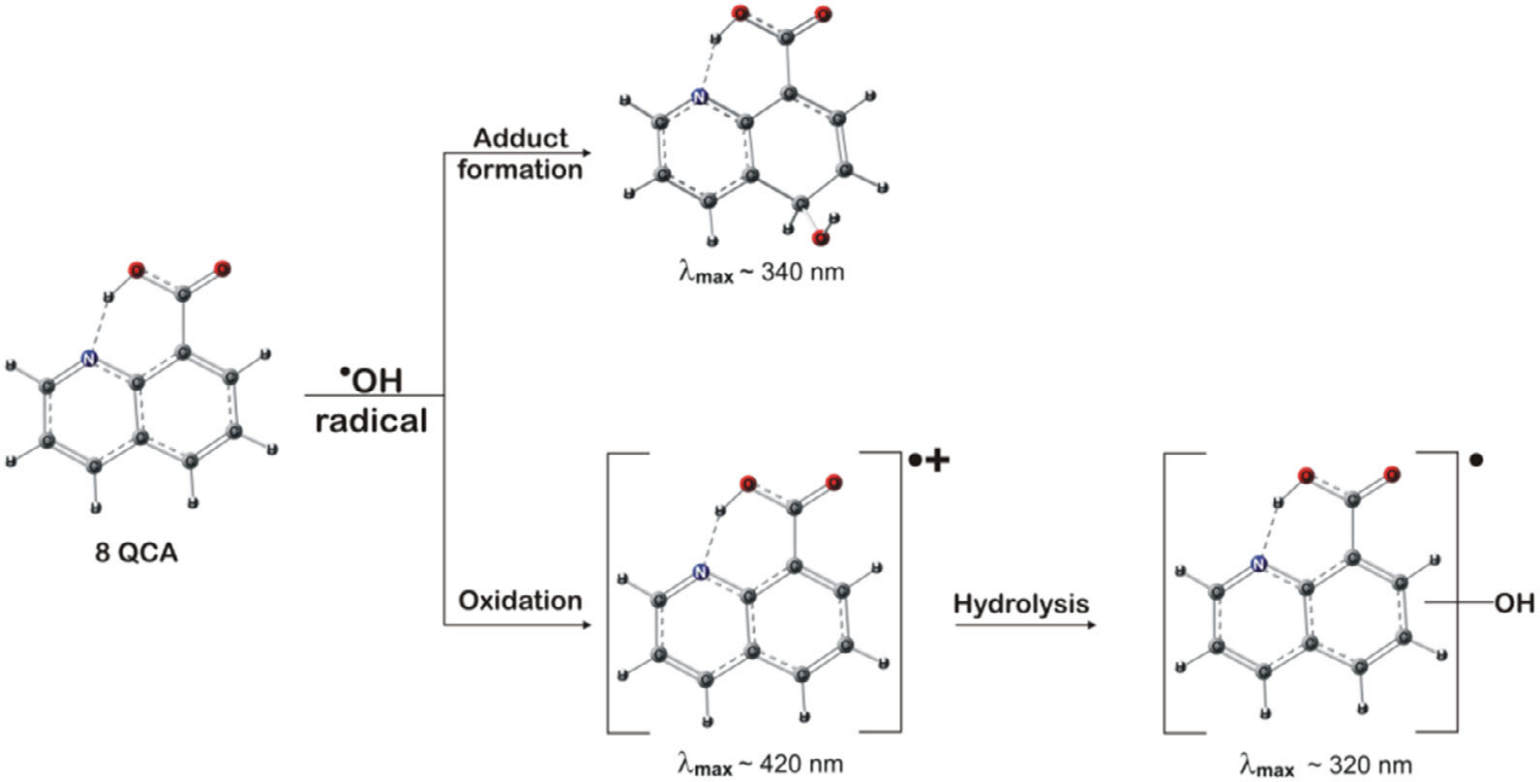
Plausible hydroxyl radical induced reactions of 8QCA with tentative spectroscopic characterization.

To decipher the reason for such pH‐dependent behavior, the absorbencies at 320 nm and 340 nm were recorded at a function of pH (3–9.7). Such plot can be seen in **Figure** **4**. In this pH range, the *G* value of ^•^OH radical in N_2_O saturated aqueous solution is fairly constant, and from the results, it was observed that the absorbance at 320 nm increased with increase in pH, while that at 340 nm did not show any pH dependent. The sigmoidal fit of the absorbance value against pH gave an inflection point at 6.4, which is very close to the ground state pKa of 8QCA. From this, it can be inferred that at acidic pH, ^•^OH radical may react with 8QCA predominantly by forming OH‐adduct, while as the pH increases, some fraction of ^•^OH radical may oxidize the 8QCA to form the radical cation. The overall reaction mechanism is depicted in **Scheme** **2**.

### 2.3 Reactions of $SO_{4}^{•-}$ Radical Anion with 8QCA

**Figure 5:**
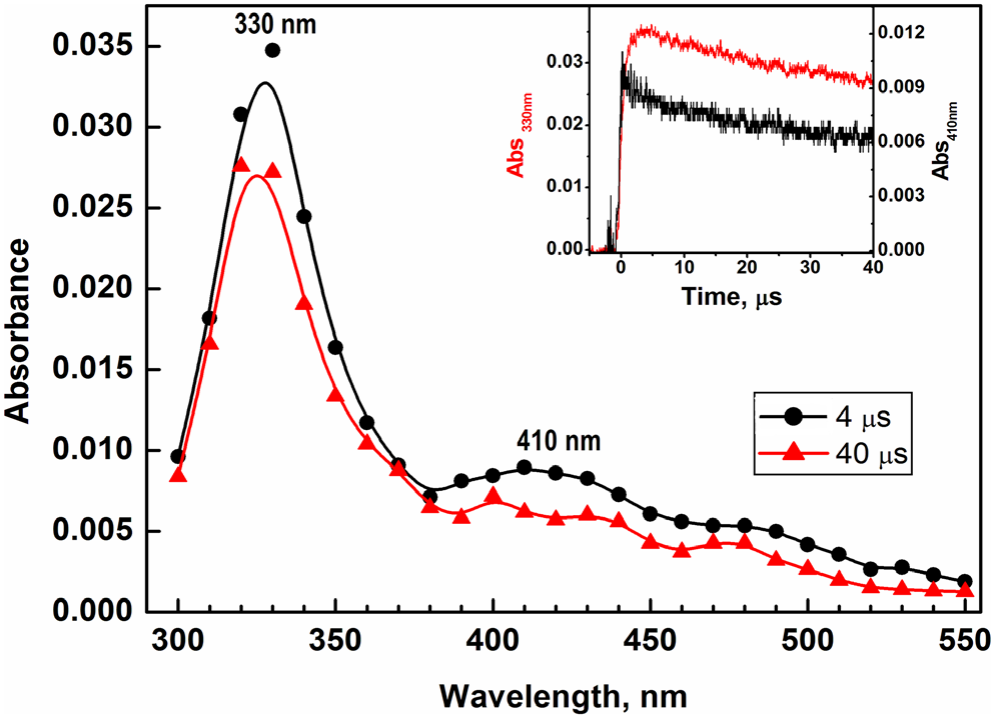
The transient absorption spectra observed at 4 and 40 μs after the pulse in the reactions of ${\text{SO}}_{4}^{•-}$ with 8QCA (1 mM) at pH 6.8 (buffered with phosphate). Inset: The absorbance‐time plot at 330 nm (red) and 410 nm (black).

As ^•^OH radical reacts with different pathways with organic substrate, strong one‐electron oxidant like sulfate radical anion, ${\text{SO}}_{4}^{•-}$, was utilized to ascertain the nature of the transient formed. As seen in **Figure** **5**, the reaction between 8QCA and ${\text{SO}}_{4}^{•-}$ resulted in the formation of transient absorption spectrum with maximum of 330 nm with very weak and broad absorption in the region 370−500 nm with a small peak at 410 nm. The bimolecular rate constant for the reaction between 8QCA and ${\text{SO}}_{4}^{•-}$ was estimated by following the buildup kinetics at 330 nm and was estimated to be 1.4 × 10^9^ M^−1 ^s^−1^ at pH 7. The lower rate constant observed in 8QCA than that reported for pyridine and quinoline derivatives may be attributed to the presence of the negatively charged carboxylate group. The transient absorbing at 330 nm decayed by following first‐order kinetics with the rate of 7.0 × 10^3^ s^−1^, from which the half‐life of the species is calculated to be 99 μs. The radical cation in quinoline is reported to undergo facile hydrolysis to form OH‐adduct, while certain quinoline carboxylate is reported to undergo decarboxylation followed by H atom shift. In the former condition, the transient will show reactivity with oxygen while under the latter condition, the transient will not show any reactivity with oxygen. To understand the fate of the radical cation formed in 8QCA, the decay of the absorbance was investigated in the presence of varying concentrations of oxygen. By following the time‐dependent change in absorbance at 320 nm in the presence of varying concentration, the bimolecular rate constant with oxygen was estimated to be 2.6 ± 0.3 × 10^7^ M^−1 ^s^−1^. Further, compared to the transient formed by ^•^OH radical, the absorption band of the 330 nm appears to be narrower. The results indicate that the ^•^OH radical can react nonspecifically with 8QCA at different positions to form different cyclohexadienyl radical which have overlapping absorbance. As seen from the time absorbance plot in Figure 5, the absorbance time plots of 330 and 410 nm are completely different citing it to be a different type of transient. Further, a closure look at the decay profile of the transient absorbing at 410 nm is found to decay faster as compared to 330 nm. From this observation, it can be inferred that the transient absorbing at 410 nm may be attributed to the formation of the radical cation of 8QCA.

### 2.4 Reaction with Hydrated Electron $\left(e_{aq}^{-}\right)$

**Figure 6:**
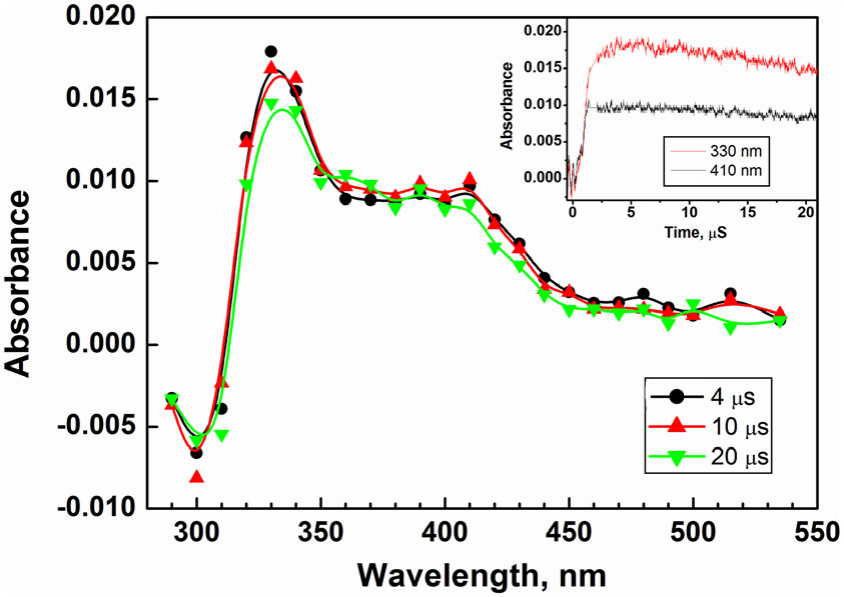
The transient absorption spectra observed at 4 and 20 μs after the pulse in the reactions of the reaction between $e_{\text{aq}}^{-}$ with 8QCA (0.1 mM) at pH 6.8 (buffered with phosphate). Inset: The absorbance‐time plot at 330 nm (red) and 410 nm (black).

The reaction of 8QCA with $e_{\text{aq}}^{-}$ was studied at pH 7 by following the decay of the $e_{\text{aq}}^{-}$ at its characteristic absorbance at 700 nm. Under the experimental condition, $e_{\text{aq}}^{-}$ decay with first‐order kinetics (4 × 10^5^ s^−1^). The decay was observed to become faster in the presence of 8QCA and the pseudo‐first‐order rate constant increased linearly with 8QCA concentration. The slope of this linear plot gave a value of bimolecular rate constant of 5.6 × 10^9^ M^−1^ s^−1^. As seen in **Figure** **6**, the transient absorption spectrum generated on reaction of 8QCA with $e_{\text{aq}}^{-}$ spanned from 300 to 540 nm with maxima at 330 nm and broad band in the region 370–410 nm. Comparing the absorption time plot of these two absorbance bands showed that the absorbance growth at 410 nm is faster and completed within 2 μs after the pulse, while that of 330 nm took ≈6 μs to complete. Moreover, the decay time for the absorbance at 410 nm differed significantly from the formation time scale of the transient absorbing at 330 nm. This suggests that these two absorbances originate from different transient species. This indicated the origin of these absorbencies stems from different natures of transient. The transient decayed by following first‐order kinetics with decay rate constant of 4 × 10^3^ s^−1^. Similar results were observed in the pulse radiolysis of 8–hydroxyquinoline–5–sulphonic acid (HQ), where the reducing radical reacted slowly with the singly negatively charged HQ to form a transient absorbance at 340 nm.^[^ ^44^ ^]^ In contrast, the singly positively charged HQ reacted with the reducing radical at a diffusion‐controlled rate, forming a transient absorption at 440 nm. The broad transient absorption spectrum observed with 8QCA further supports the idea that the two transient species are formed due to the existence of different species in prototropic equilibrium. A detailed theoretical calculation of these species is discussed in the later section.

**Scheme 3:**
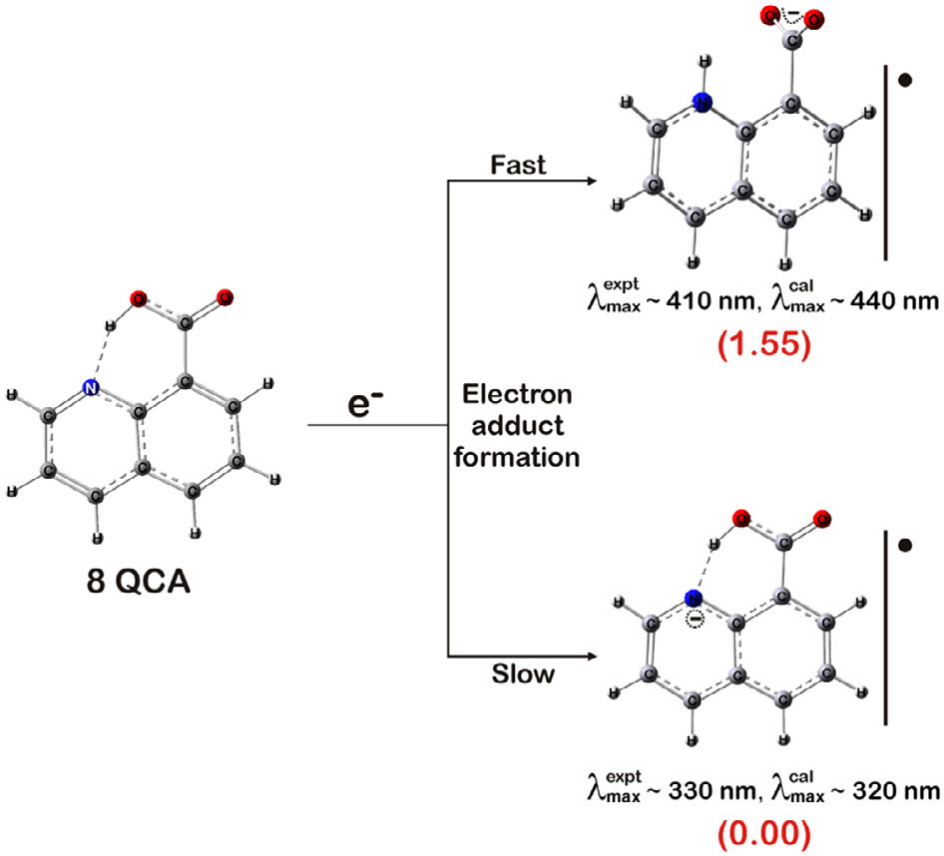
Plausible hydrated electron $\left(e_{aq}^{-}\right)$‐induced reactions of 8QCA with tentative spectroscopic characterization. The value in red color inside the bracket shows the relative energy of these species (for details, see text).

The overall reaction hydrated electron $\left(e_{aq}^{-}\right)$ can be depicted in **Scheme** **3**.

### 2.5 Degradation Studies

**Figure 7:**
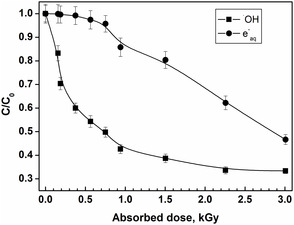
Plots of the ratios of residual concentration (*C*) to initial concentration (*C*_O_) of 8QCA obtained at different absorbed doses using ^•^OH radical (▪) and $e_{\text{aq}}^{-}\;(•)$ at pH 7.

The degradation studies of 8QCA were investigated under steady‐state radiolysis using γ radiation. Here, 100 μM 8QCA was irradiated in a condition to generate ^•^OH radical. As seen from the Figure S3, S4, Supporting Information, the absorbance band at 230 nm was found to decrease monotonically with an increase in absorbed dose, with an increase in the absorbance at 375 nm. Similarly, the degradation of 8QCA under the reducing condition was also monitored. Here also along with other changes in the absorption spectrum, the band at 230 nm was found to decrease with increase in absorbed dose. Thus the absorption band at 230 nm was evaluated further for comparing the degradation studies. The degradation kinetics of 8QCA in the presence of ^•^OH radical and $e_{\text{aq}}^{-}$ as a function of absorbed dose can be seen in **Figure** **7**. Here, the ratio of C_o_ and C, the initial and residual 8QCA concentration after γ irradiation, respectively exhibited exponential decrease in 8QCA concentration with absorbed doses. Also from the degradation, under the applied experimental conditions, the highest degradation rates of 8HQC were observed with ^•^OH radical. In this case, one can argue that the *G* value of ^•^OH radical (0.6 μmol J^−1^) is higher as compared to the total amount of radiolytically produced $e_{\text{aq}}^{-}$ (0.28 μmol J^−1^), resulting in increase in the degradation rate. Therefore, the degradation efficiency of the radical was estimated by taking the ratio of the *G*_(−8QCA)_ with *G* value of the primary radical. The ratio at ≈150 Gy was 0.17 and 0.002 for ^•^OH radical and $e_{\text{aq}}^{-}$, respectively, indicating that 1 μmole of ^•^OH radical and $e_{\text{aq}}^{-}$ degrades 0.17 and 0.002 μmoles of 8QCA.

Further to get an understanding of the reaction mechanism, the nature of the transient is assigned with the help of quantum chemical calculations.

## 3 Computational

**Figure 8:**
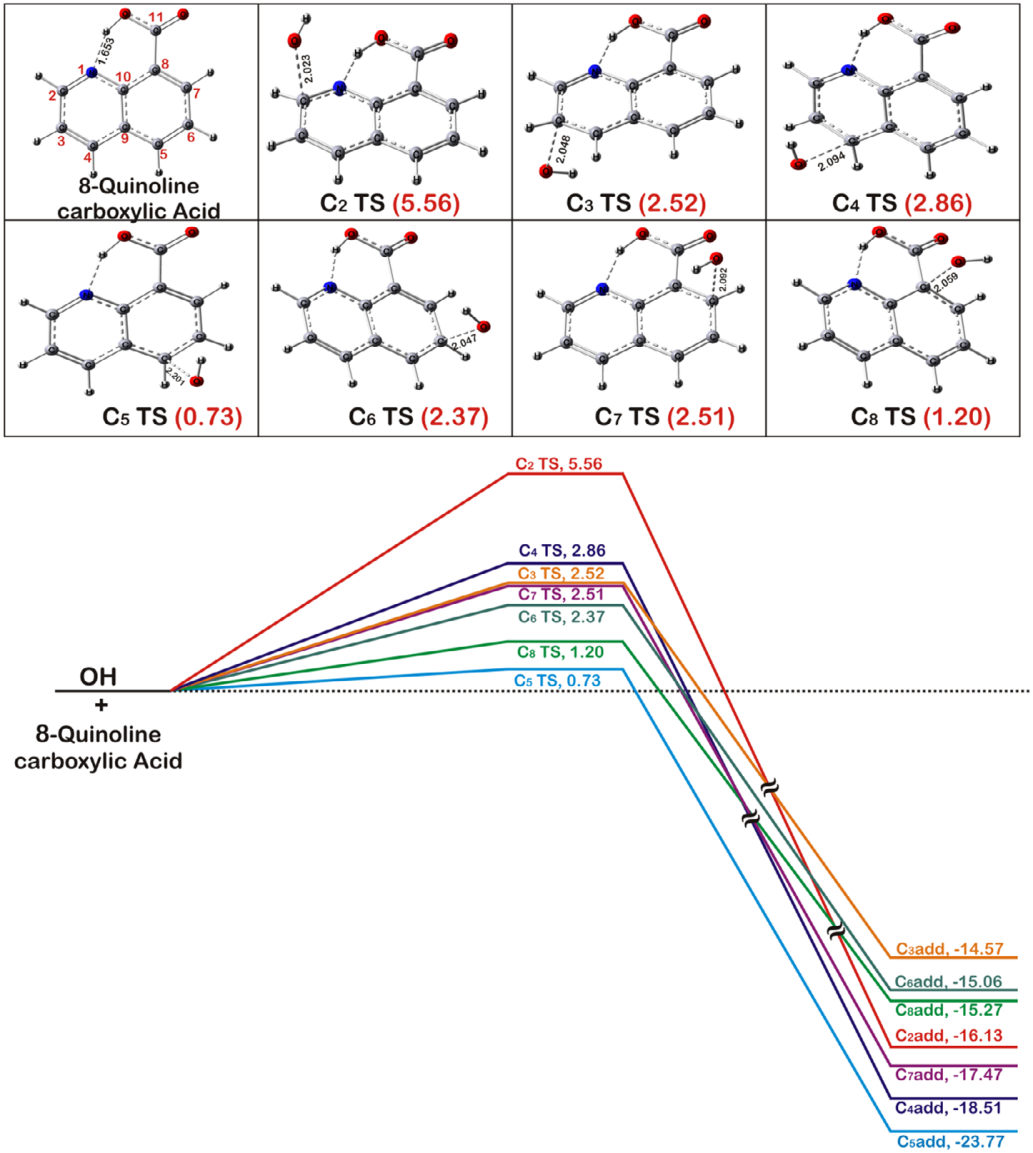
Optimized geometries (length in Å) of various TSs in the different channels of the ^•^OH radical addition reaction with neutral quinclorac at B3LYP/6–311+G(d,p) level of theory incorporating the solvent effect. Lower panel shows relative energy (kcal mol^−1^) diagram calculated at B2PLYP–D level of theory with modified variants of aug–cc–pVTZ basis set at the above geometries including ZPE correction (for details, see text).

The optimized structure of the most stable, lowest energy conformer of neutral 8QCA at B3LYP/6–311+G(d,p) level of theory can be seen in **Figure** **8**. The structure is planar with a strong H‐bond between H atom of the —COOH moiety and the nitrogen atom of quinoline ring. Generally, 8QCA has higher energy conformer owing to the hydrogen bonding involving H atom of the —COOH moiety with the nitrogen atom of quinoline ring. In such case, the most stable conformer was taken for the present study which has a planar geometry with a strong —COOH^……^N hydrogen bonding.

### 3.1 pKa Calculation

The pKa value of the 8QCA is determined using the theoretical method also. In the present study, the second pKa is evaluated using protocol based on a direct approach as described in the literature.^[^ ^45^, ^46^ ^]^ One water molecule is considered for the explicit solvation purpose. DFT calculations were performed in the presence of one explicit water molecules using M062x functional with 6‐311 G+(d,p) basis set and a solvation model based on density was used. Single point energy is calculated at B2PLYP–D level of theory with modified variants of aug–cc–pVTZ basis set for accurate energy determination. The pKa value thus determined is found to be ≈6.7 as compared to the experimental value of 7.2. The result shows an excellent agreement considering the level of theory used. Details can be seen in Scheme S2, Supporting Information. Moreover, the excellent agreement between the experimental and calculated pKa values further validates the present theoretical methods used.

### 3.2 Rate Constant Determination

It is well known in the literature that the ^•^OH radical addition reaction into the bridged carbon atom (C_9_ and C_10_) is very slow.^[^ ^40^, ^41^ ^]^ Hence, seven numbers of carbon atoms which are present in the ring, namely, C_2_ to C_8_, have been studied for the ^•^OH radical addition reaction. The major reaction channel in the ^•^OH addition is the formation of OH‐adduct in the case of unsaturated hydrocarbons.^[^ ^47^, ^48^ ^]^ Various TS structures that originate in the addition reaction of ^•^OH radical with different carbon atoms (C_2_ to C_8_) in neutral 8QCA can be seen in Figure 8. These TSs are named as “C_n_ TS,” where “n” denotes and numbering of carbon atom. In the next few paragraphs, a brief description of these structures will be discussed. Almost all the TSs behave like a loose reactant‐like structure with a low imaginary frequency ranging from 150 cm^−1^(C_5_TS) to 375 cm^−1^(C_2_TS). Similarly, the C—OH bond distance in various TSs varies from 2.023 Å (C_2_TS) to 2.201 Å (C_5_TS) and can be seen in Figure 8. Furthermore, a nice correlation was observed between the C—OH bond distance and the magnitude of imaginary frequency of the TSs. The effective activation barrier height for these TSs varies from a minimum value of 0.73 kcal mol^−1^ (C_5_TS) to a maximum value of 5.56 kcal mol^−1^ (C_2_TS). All these parameters can be seen in Figure 8 as well. This confirms that the most reactive site for the OH radical reaction in neutral 8QCA is C5. Two carbon atoms positioned at 8 and 5, namely, C8 and C5, show a low effective barrier height.

As discussed in earlier section, the single point energy is calculated at B2PLYP‐D level of theory which takes care of any dynamic correlation present in the system. However, the B2PLYP‐D method does not make any correction for the nondynamic correlation. In such case, it is useful to know the ratio of single/multireference (SR/MR) nature of the molecular wavefunction. One such widely used indicative tool is the T1 diagnostic which is obtained from CCSD calculation. To assess the error in the energy calculation due to nondynamical correlation or the MR character, T1 diagnostic calculations were performed for the TS with the lowest barrier, that is, C_5_TS at CCSD/6–311g(d) level of theory. In the present study, the T1 diagnostic for C_5_TS is calculated as 0.0216. In a detailed study by Lee and Taylor, it has been suggested that the SR methods perform well for molecules with a T1 diagnostic value smaller than 0.02.^[^ ^49^ ^]^ In the present case, the calculated value of 0.0216 is comparable to the suggested limit value of 0.02. This clearly suggests that the nondynamical correlation can be neglected in the present system. The above effort suggests that the B2PLYP‐D method can be safely used for the single‐point energy calculation in the present system.

The details for rate constant calculation using deformed transition state theory (d‐TST) method can be seen in our previous study for ^•^OH radical reaction.^[^ ^41^, ^50^ ^]^ To see the contribution of the tunneling correction in the present bimolecular reaction, rate constants were also calculated with conventional TST. A detail comparison between these two methods, namely, TST and d‐TST, used for calculation of the rate constants in the ^•^OH radical addition reaction for various carbon atoms can be seen in the Table S2, Supporting Information. The difference observed was very negligible. Generally, the tunneling is very much pronounced in light atom (H atom) transfer/abstraction reaction and also at low temperature. In the present case, the ^•^OH radical reaction proceeds mainly through an addition mechanism forming OH–adducts. Hence, the tunneling correction was negligible in the present case. Once the reaction passes through the respective TSs, an OH–adduct is formed. Structures of various OH–adducts can be also seen in the Figure S5, Supporting Information. Similar to the nomenclature of the TSs, the OH–adduct has been named as “C_n_ add,” where “C_n_” stands for the number of carbon atom and “add” is for the adduct. All the OH–adducts are highly stabilized with stabilization energy ranging from 14.57 kcal mol^−1^ (C_3_ add) to 23.77 kcal mol^−1^ (C_5_ add). Optimized structure for various adducts with their relative energy can be seen in Figure S5, Supporting Information. The absorption spectra for various adducts were also calculated theoretically to compare with the experimentally obtained transient spectra. The calculated *λ*_max_ value of “C5 add” OH adducts matches very well with the experimentally observed value which confirms that the most reactive site is C5 carbon atom. Moreover, the stabilization energy (23.77 kcal mol^−1^) also suggests that the most stable adduct is the C5–OH adduct. **Table** **1** shows the effective barrier height ($\varepsilon^{‡}$), Gibbs energy of activation (Δ$G^{‡}$), and the respective observed rate constants for various carbon atom calculated theoretically for neutral 8QCA.

**Table 1:** Effective barrier height ($\varepsilon^{‡}$), Gibbs energy of activation (Δ$G^{‡}$), and the respective observed rate constants for various carbon atoms calculated theoretically for neutral 8QCA.

	$\varepsilon^{‡}$ [kcal mol^−1^]	Δ$G^{‡}$ [kcal mol^−1^]	$k_{\text{obs}}$ [M^−1 ^s^−1^]
C2	5.56	14.06	4.44 × 10^5^
C3	2.52	11.14	6.06 × 10^7^
C4	2.86	11.34	3.96 × 10^7^
C5	0.73	8.94	1.45 × 10^9^
C6	2.37	10.93	8.28 × 10^7^
C7	2.51	10.97	7.00 × 10^7^
C8	1.20	10.05	3.47 × 10^8^

### 3.3 Local Reactivity Parameter

**Figure 9:**
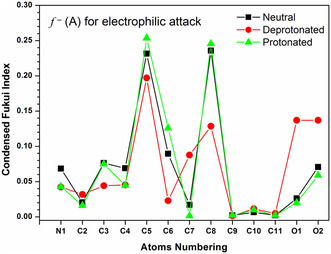
Condensed Fukui index (*f* ^−^) for various atoms in different species of 8QCA for electrophilic attack. Atom numbering can be seen in Figure 8.

The computational study can also be exploited to characterize the site‐specific or local reactivity parameter for the ^•^OH radical toward each carbon atoms of 8QCA. As discussed in methodology section, condensed Fukui functions were evaluated for this purpose for protonated, neutral, and anionic forms of the 8QCA for each atom and can be seen in **Figure** **9**. The details of these calculations can be seen in our recent publication.^[^ ^50^, ^51^ ^]^ The outcome of such study for the electrophilic addition of ^•^OH radical toward each carbon atom shows that the carbon atoms present in the ring containing nitrogen atom are less reactive toward the ^•^OH radical as compared to the carbon atoms present in the ring containing —COOH group.

The outcome of the study also shows that the protonated, neutral, and anionic species behave similarly toward the electrophilic attack of ^•^OH radical. The carbon atom at 5^th^ position (C5) shows a maximum reactivity followed by C8 toward the ^•^OH radical reaction for protonated, neutral, and anionic species. The observation of reactivity trend using CFF matches very well with the effective barrier height calculation and in fact they are directly correlated for protonated and neutral species. However, for anionic species, the C8 carbon atoms show low barrier (see Table S1, Supporting Information) as compared to C5 carbon atom suggesting that in anionic species the C8 carbon atom is more reactive as compared to C5 carbon atom. This trend contradicts the result of CFF as shown in Figure 8. The reason for this discrepancy lies in the TS structure of C8 which is stabilized through strong hydrogen bond between the ^•^OH radical and the anionic —COO^−^ moiety. Once the most reactive site is confirmed, the rate constant for ^•^OH radical with protonated and anionic species is also calculated theoretically for C5 and C8 carbon atoms. The rate constant for C5 and C8 carbon atoms in protonated and deprotonated species with ^•^OH radical shows a higher value as compared to the neutral species. The effective barrier height $\varepsilon^{‡}$ in the ^•^OH radical reaction for the protonated and anionic species is always lower as compared to the neutral species which indicates that the protonated and anionic species are more reactive as compared to the neutral one (Table S1, Supporting Information).

### 3.4 Nature of Radical Anion [8QCA]^**•−**^

To shed more light on the nature of the radical anion adduct of 8QCA, theoretical calculations have been performed. The structure of radical anion [8QCA]^•−^ is optimized using a similar method as described in the main text. While optimizing the structure it has been observed that the proton transfer process is taking place from the —COOH group to the N moiety in the quinoline ring giving rise to another structure for [8QCA]^•−^ which is protonated at N site. The energy difference between these two isomers is calculated at B2PLYP‐D/modified version of aug‐cc‐pVTZ level of theory and estimated to be ≈1.55 kcal mol^−1^. This suggests that radical anion [8QCA]^•−^ can exist in two different forms at room temperature. The UV‐vis absorption spectra for these isomers are calculated using TD‐DFT method at CAM‐B3LYP/aug‐cc‐pVDZ level of theory. The *λ*_max_ value for the normal H–bonded radical anion [8QCA]^•−^ is calculated to be ≈320 nm. In this species, the charge is mostly localized at N site. Similarly, the *λ*_max_ value for the protonated radical anion [8QCA]^•−^ is calculated to be ≈440 nm. In this case, the charge is mostly localized around –COO^−^ group (Scheme 3). These calculated absorption values nicely match the experimentally observed absorption values for these two radical anionic species of 8QCA.

## 4 Conclusions

In summary, experimental and computational methods have been employed to study the kinetic and mechanistic aspects of the hydroxyl radical (^•^OH), sulfate radical anion (${\text{SO}}_{4}^{•-}$), and hydrated electron $\left(e_{\text{aq}}^{-}\right)$ reaction with quinoline‐based herbicide, namely, 8QCA in aqueous media. The pulse radiolysis technique has been used to perform these studies at different pHs and results are corroborated with theoretical studies using ab initio molecular orbital (MO) calculations. The reaction of ^•^OH radical shows pH‐dependent behavior. At lower pH of 1, 8QCA exists as singly positively charged due to the protonation which reacts with ^•^OH radical to generate transient spectrum showing absorption maxima at 340 and 420 nm. Similarly, a higher pH of 9, where 8QCA is deprotonated and exists as anionic species, shows a transient absorption spectrum with maximum of 320 nm. At neutral pH 0f ≈7, it exists as neutral species and reacts with ^•^OH radical differently. The absorbance changes at 320 nm were pH dependent, while that at 340 nm was independent of the pH of the solution. The experimentally evaluated bimolecular rate constant of ^•^OH radical, ${\text{SO}}_{4}^{•-}$ radical anion, and $e_{\text{aq}}^{-}$ with 8CCA at neutral pH where it exist as neutral form is evaluated as 6.2 × 10^9^, 1.4 × 10^9^, 5.6 × 10^9 ^M^−1 ^s^−1^, respectively. Individual rate coefficients for ^•^OH radical addition reaction with each carbon atoms were evaluated using conventional TST using 1D tunneling corrections. The solvent effect on reaction is implemented through Collins–Kimball formulations. Both the approaches, namely, the Fukui index and individual rate constant determination confirm that the most reactive site for the ^•^OH radical addition in the herbicide is the C5 carbon atom which is at *para* position to the —COOH group. The total rate constant for the ^•^OH radical reaction with both neutral and anionic forms is relatively high and equal to its diffusion‐limit value and a nice match has been observed with the experimental value. The $e_{\text{aq}}^{-}$ was found to react with 8QCA to form semi reduced entity with transient absorbing at 330 and 410 nm. The ability of ^•^OH radical to degrade 8QCA was compared to $e_{\text{aq}}^{-}$ and found to be higher.

## 5 Experimental Section

5.1

5.1.1

##### Chemicals

8QCA (98%) was obtained from Sigma–Aldrich Chemical Co. and used without further purification. Other chemicals, such as buffers, were procured from Thomas Baker in high purity. High‐purity N_2_, N_2_O, or O_2_ gas was used as‐is. Aqueous solutions were prepared using water purified with a Millipore Milli‐Q system. Steady‐state absorption studies were performed on a Shimadzu 650 absorption spectrophotometer. The pH of the solution was maintained using buffer solutions prepared from Na_2_HPO_4_ and NaH_2_PO_4_ mixtures, with pH adjustments made using HClO_4_ or NaOH.

##### Experimental

Pulse radiolysis studies were performed by using a linear accelerator delivering 7 MeV electron pulses with a pulse width of 200 ns coupled to an optical absorption detection set‐up. Details of the facility can be seen elsewhere.^[^ ^52^ ^]^ Transient species were monitored using the optical absorption detection method. To determine the dose per pulse, an aerated KSCN solution (0.01 M) was used to monitor the formation of ${\text{SCN}}_{2}^{•-}$ at 475 nm, using a value of 2.59 × 10^−4^ m^2^ J^−1^ for *G* × *ε* at 475 nm.^[^ ^53^ ^]^ The radiation chemical yield, *G*, is the amount of product formed upon absorption of 1 J of energy (mol J^−1^), while *ε* is the molar absorption coefficient (m^2^ mol^−1^). The dose was maintained at a range of 10–12 Gy/pulse, where 1 Gy is equal to 1 J kg^−1^. When electron pulses irradiate deaerated aqueous solutions, water undergoes radiolysis through a series of complex reactions initiated by ionization and excitation. These processes lead to the production of free radicals and molecular products. On a picosecond timescale, these reactive species diffuse throughout the solution, and their formation over this period is described by Equation (1). Since the reactions between the radical species and 8QCA in this study are investigated on timescales greater than 1 μs, only the radiolytic products formed after the diffusion of the initial spur are relevant. Notably, with low LET radiation at pH 7, among the radical species generated, the *G* of hydroxyl radical (^•^OH, 0.28 μmol J^−1^) and hydrated electron ($e_{\text{aq}}^{-}$, 0.28 μmol J^−1^) are significantly higher compared to that of hydrogen atom (^•^H, 0.06 μmol J^−1^). Details of various processes and reaction occurring during the water radiolysis can be seen in the literature^[^ ^54^ ^]^ and also included in Scheme S1, Supporting Information.
(1)

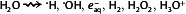




The ^•^OH radical exhibits oxidizing properties, while $e_{\text{aq}}^{-}$ is a strong reducing species. To ensure reactions of ^•^OH solely with substrate molecules, the $e_{\text{aq}}^{-}$ generated during the radiolysis is quantitatively converted to ^•^OH by saturating the system with N_2_O‐gas (Equation (2)). Due to higher solubility of N2O, some fractions of $e_{\text{aq}}^{-}$ are scavenged in the spur, and under these reaction conditions, the *G* of ^•^OH is estimated to be 0.60 μmol J^−1^.
(2)

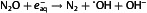




In addition to the ^•^OH, oxidations of 8QCA have been conducted using radiolytically generated sulphate radical anion (${\text{SO}}_{4}^{•-}$). The ${\text{SO}}_{4}^{•-}$ was generated by radiolyzing N_2_ saturated aqueous solution containing 10 mM peroxydisulfate and 1% tert‐butanol. Reaction with $e_{\text{aq}}^{-}$ was studied by radiolysis of deaerated aqueous solution containing 1% v/v tert‐butanol and 8QCA at pH 1.

Steady‐state radiolysis was performed using ^60^Co Gamma Chamber having activity of 5000 Ci (model 1200, BRIT make). The dose rate as determined by the Fricke dosimeter was found to be 188 Gy min^−1^. In a typical degradation experiment, aqueous solutions of 8QCA at initial concentration of 100 μM were treated with the condition to exclusively generate ^•^OH radical or $e_{\text{aq}}^{-}$. The solution was subjected to absorbed doses ranging from 0.15 to 3.0 kGy and the amount degraded was calculated by dividing the differential absorbances between the unirradiated and irradiated solution with the extinction coefficient value of 23 107 M^−1^ cm^−1^ and 23 685 M^−1^ cm^−1^ at 230 nm for solution in absence and presence of 1% v/v tert‐butanol at pH 7, respectively. Degradation experiments were performed in triplicate and the error bars on the figures represent the mean ± SD of the replicates.

The radiation‐chemical yield of 8QCA degradation (*G*_–8QCA_, μmole J^−1^), expressed as the amount degraded (ΔC, μmol L^−1^) per unit of absorbed dose (D, Gy) in water assuming density of 1 gm cm^−3^, was calculated by following Equation (3)

(3) $$G_{–8\text{QCA}}=\frac{\Delta C}{\text{Dose}}$$

An indicator of the efficiency of each radical in degrading 8QCA can be expressed as the *G*‐value of 8QCA formed per *G* value of the radical.

##### Computational

The ^•^OH radical addition reaction channels for various carbon atoms in 8QCA were explored by generating their respective potential energy surfaces using ab initio MO calculations. The geometry optimization process of 8QCA in the ground electronic state including all the intermediates and transition states was performed using Becke three‐parameter hybrid functional with nonlocal correlation provided by the Lee–Yang–Parr (B3LYP) expression employing 6–311+g(d,p) basis set followed by their respective frequency calculation.^[^ ^55^ ^]^ This also provides the zero‐point energy (ZPE) correction. In the optimization process of all the species, the solvent effect was also introduced using the polarizable continuum model with water as solvent. Structure minima or the transition state was confirmed with their number of imaginary frequencies (negative eigenvalue) which is either 0 or 1. Reaction pathways for various reactions were established by running intrinsic reaction coordinate calculations. The energy of these various structures was then refined by performing the single point energy calculation at higher level of theory using a hybrid density functional with perturbative second‐order MP2 correction known as B2PLYP–D method^[^ ^56^, ^57^ ^]^ employing modified variants of aug–cc–pVTZ basis set.^[^ ^58^ ^]^ In our earlier study, it has been shown that the B2PLYP–D method has good reproducibility in some molecular system and takes care of any error in energy calculation arising from the dynamic electron correlation.^[^ ^59^ ^]^ In the present study, the <S^2^> values for various open shell structures were well within the expected value showing no spin contamination. Local reactivity parameters (LRP) were evaluated in terms of HSAB principle using the DFT methods by calculating Fukui indices using natural population analysis.^[^ ^60^, ^61^, ^62^ ^]^ The details of these calculations can be seen in the recent publication.^[^ ^41^, ^50^, ^51^ ^]^ All of the calculations were performed using mainly the GAMESS quantum–chemistry suite and the Gaussian 09 package of programs.^[^ ^63^, ^64^ ^]^

The reaction rate constant of ^•^OH radicals with 8QCA has been calculated using formulations based on the conventional TST for a general bimolecular reaction, such as Reactants → $TS^{‡}$ → Products. The details of these methods and assumptions can be found in the recent publications.^[^ ^41^, ^65^, ^66^ ^]^ In the present study, the d‐TST which was first proposed by Carvalho‐Silva et al.^[^ ^67^ ^]^ including the tunneling effect has been adopted as given below

(4) $$k_{d-\text{TST}}=\frac{k_{B}T}{h}\frac{Q^{‡}}{Q_{1}Q_{2}}{\left(1-d\frac{\varepsilon^{‡}}{k_{B}T}\right)}^{\frac{1}{d}},d=-\frac{1}{3}{\left(\frac{h\nu^{‡}}{2\varepsilon^{‡}}\right)}^{2}$$

where $Q_{1}$, $Q_{2}$, and $Q^{‡}$ are the partition functions of reactants R_1_ and R_2_ and of the transition state (TS), respectively. The *h* is Planck's constant, $k_{B}$ is the Boltzmann constant, and $\varepsilon^{‡}$ is the effective height of the energy barrier, duly corrected with the ZPE. To account for the tunneling effect in the reaction at particular temperature *T*, the deformation parameter, *d,* has been introduced as defined in the earlier equation. The $\nu^{‡}$ is frequency for crossing the barrier, which uniformly covers the range from classical to moderate tunneling regimes. The diffusion of reactants in the aqueous solution is introduced using steady‐state Smoluchowski rate constant, $k_{D}$, following the Collins–Kimball theory.^[^ ^68^ ^]^ Finally, the apparent rate constant, $k_{\text{obs}}$, is calculated by combining the calculated rate constant $k_{d-\text{TST}}$ and the $k_{D}$, yielding the Equation (5)

(5) $$\frac{1}{k_{\text{obs}}}=\frac{1}{k_{d-\text{TST}}}+\frac{1}{k_{D}}$$

All kinetic and associated parameters have been calculated with the Transitivity and Eringpy codes.^[^ ^69^, ^70^ ^]^ Most of the experimental literature values for the degradation studies on pollutants are available in the temperature range of 298–300 K. Hence, all the rate constant calculations are performed at 300 K for a comparison with the literature value.

## Conflict of Interest

The authors declare no conflict of interest.

## Supporting information

Supplementary Material

## Data Availability

The data that support the findings of this study are available from the corresponding author upon reasonable request.
