# Supplementary material for: Experimental and Theoretical Studies on Reaction Kinetics, Mechanism, and Degradation of Quinoline‐Based Herbicide with Hydroxyl Radical, Sulphate Radical Anion, and Hydrated Electron
Source: Chemphyschem. 2025 May 1;26(13):e202401135. doi: 10.1002/cphc.202401135 (PMC12225751; doi:10.1002/cphc.202401135)
Supplement: Supplementary file 1 — Supplementary Material [file CPHC-26-e202401135-s001.pdf]

Supplementary Information

Experimental and Theoretical Studies on Reaction Kinetics, Mechanism and Degradation of Quinoline based Herbicide with Hydroxyl Radical, Sulphate Radical Anion and Hydrated Electron

Beena G. Singh* and Hari P. Upadhyaya*

Radiation & Photochemistry Division

Bhabha Atomic Research Centre, HBNI,

Trombay, Mumbai – 400 085, India

*)Author to whom correspondence should be addressed

Electronic mail: beenam@barc.gov.in (ORCID ID: 0000–0002–1679–5456) (BGS)

haripu@barc.gov.in (ORCID ID: 0000–0002–4742–8827) (HPU)

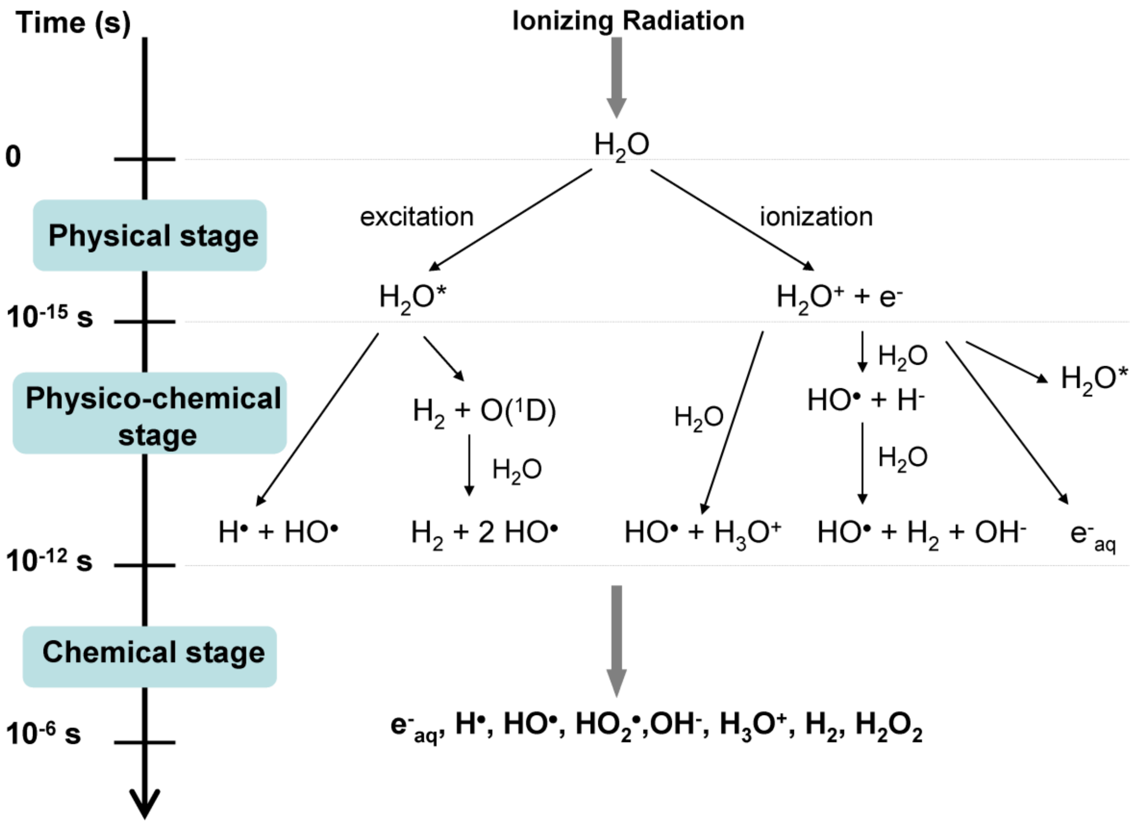

Scheme S1: Main reactions occurring during the three stages of water radiolysis. (taken from Water 2011, 3(1), 235-253; https://doi.org/10.3390/w3010235)

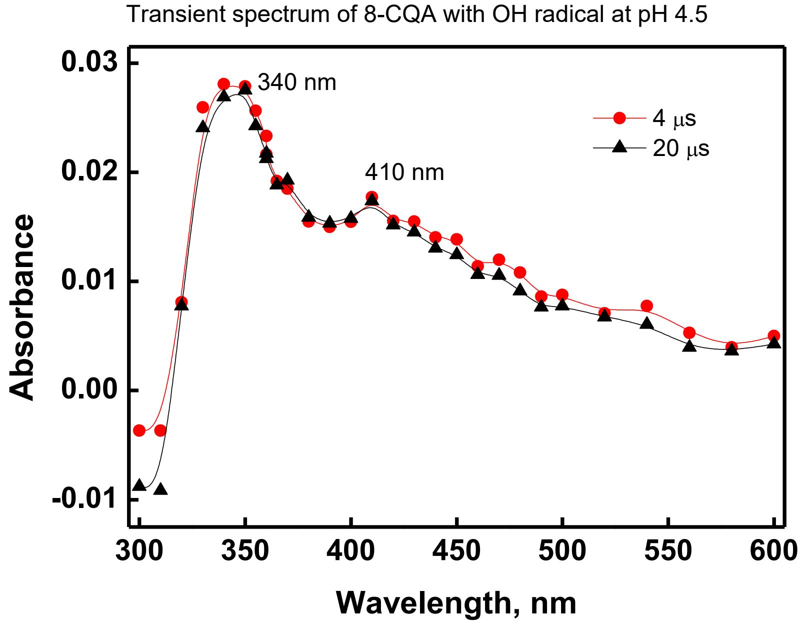

Figure S1 Transient absorption spectrum observed in N_2O saturated aqueous solution of 8QCA (1 mM) at pH 4.5

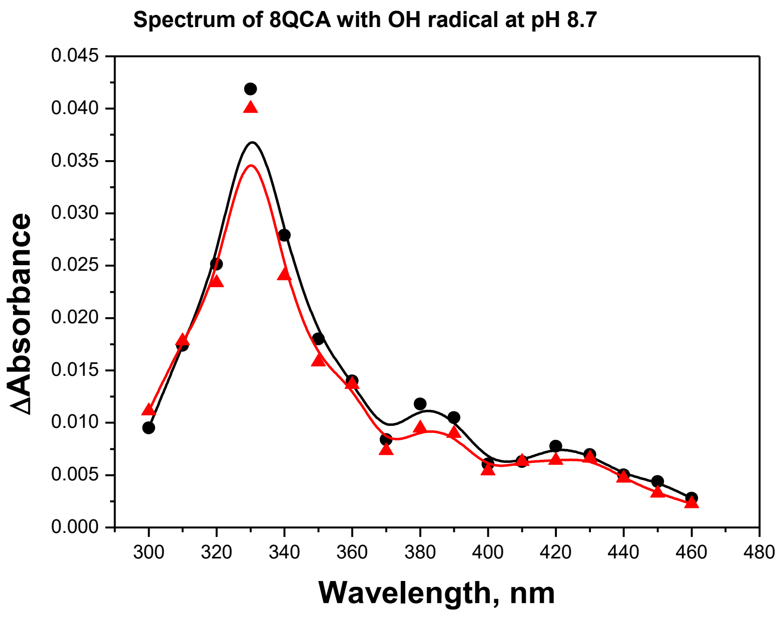

Figure S2 Transient absorption spectrum observed in N_2O saturated aqueous solution of 8QCA (1 mM) at pH 8.7.

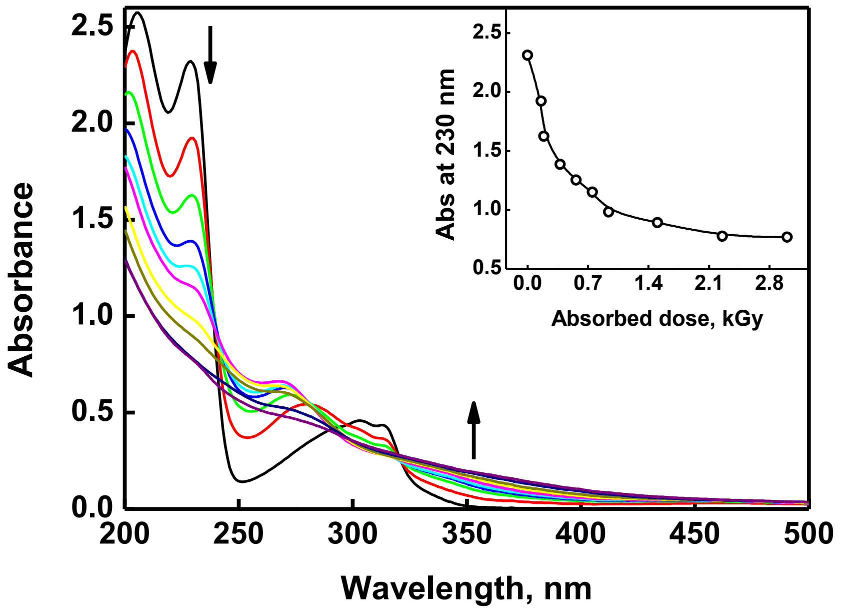

Figure S3: Absorption spectrum of N_2O saturated solution containing 8HQC (100 μM) obtained on exposure to γ radiation at different absorbed doses (0-3.0 kGy). Inset shows the decrease in the absorbance at 230 nm as a function of absorbed dose.

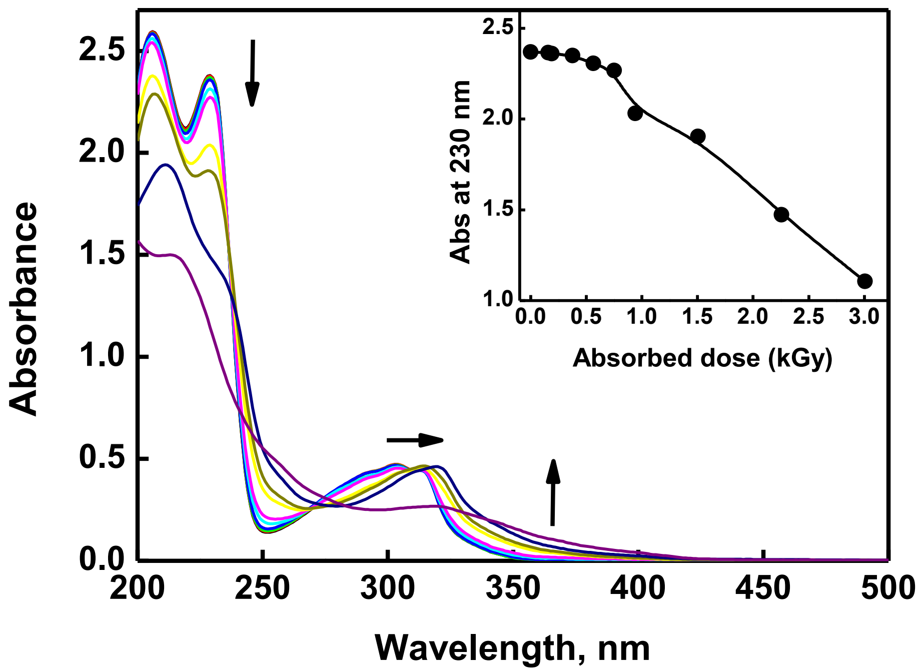

Figure S4: Absorption spectrum of deareated solution containing 8HQC (100 μM) and 1% tert-butanol (v/v) obtained on exposure to γ radiation at different absorbed doses (0-3.0 kGy). Inset shows the decrease in the absorbance of the said solution at 230 nm as a function of absorbed dose.

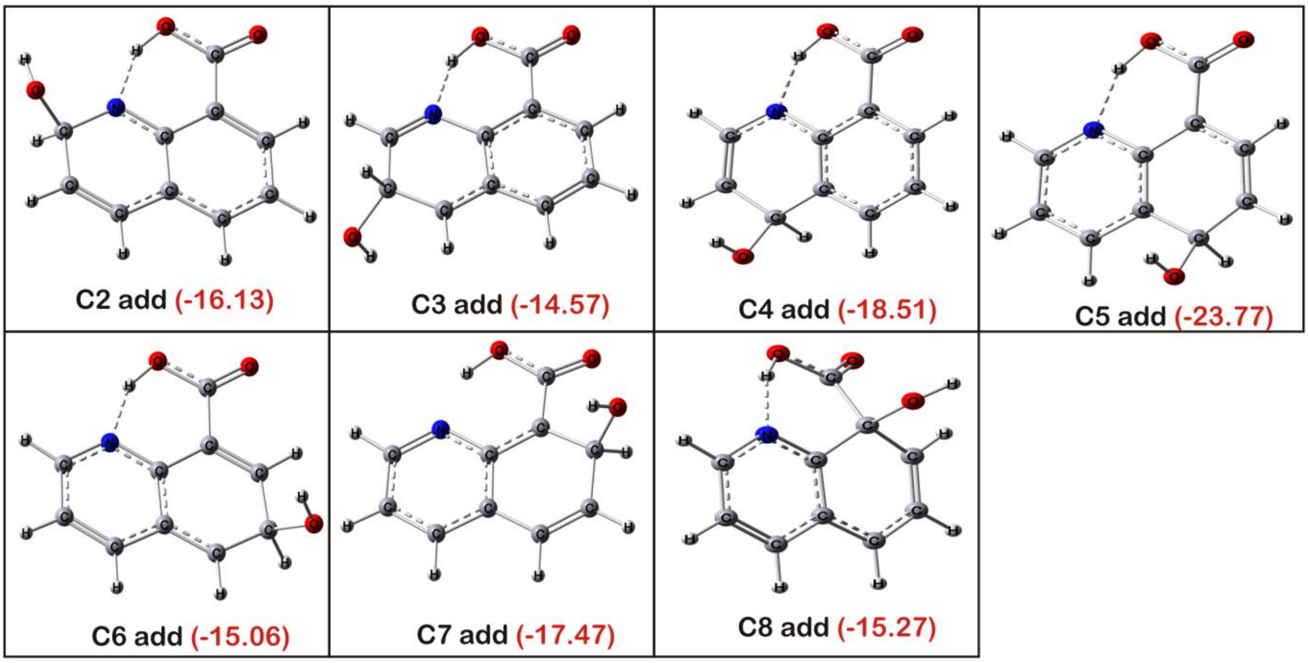

Fig. S5: Optimized geometries (length in Å) of various OH-adducts in the different channels of the OH addition reaction with neutral 8QCA at CAM-B3LYP/6–311+G(d) level of theory incorporating the solvent effect. The relative energy (kcal/mol) calculated at B2PLYP–D level of theory with modified variants of aug–cc–pVTZ basis set is shown in the bracket including ZPE correction.

Table S1: Effective barrier height for most reactive carbon atom calculated theoretically for protonated, neutral and deprotonated species of 8QCA

	$\varepsilon^{\ddagger}$ (kcal/mol)		
	Protonated (+1)	Neutral (0)	Deprotonated (–1)
C5	–0.59	0.73	0.00
C8	0.24	1.20	–0.72

Estimation of pK_a value of 8QCA using theoretical methods

In the present study, the second pKa of 8QCA is estimated theoretically based on the method described in reference (An Accurate Approach for Computational pK_a Determination of Phenolic Compounds. Molecules 2022, 27, 8590) where an accurate protocol based on a direct approach is proposed for computing pK_a of various phenols Importantly, this methodology does not require the use of correction factors or mathematical fitting, making it highly practical, easy to use and fast. DFT calculations were performed in the presence of one explicit water molecules using M062x functional with 6-311G+(d,p) basis set and a solvation model based on density (SMD) was used. One water molecule is considered for the explicit solvation purpose. Single point energy is calculated at B2PLYP–D level of theory with modified variants of aug–cc–pVTZ basis set for accurate energy determination. The pK_a value thus determined is found to be ~6.7 as compared to the experimental value of 7.2 using the following equation.

$$pK_a = \Delta E_{dep} / 2.302RT + 15.74$$

The result shows an excellent agreement considering the level of theory used.

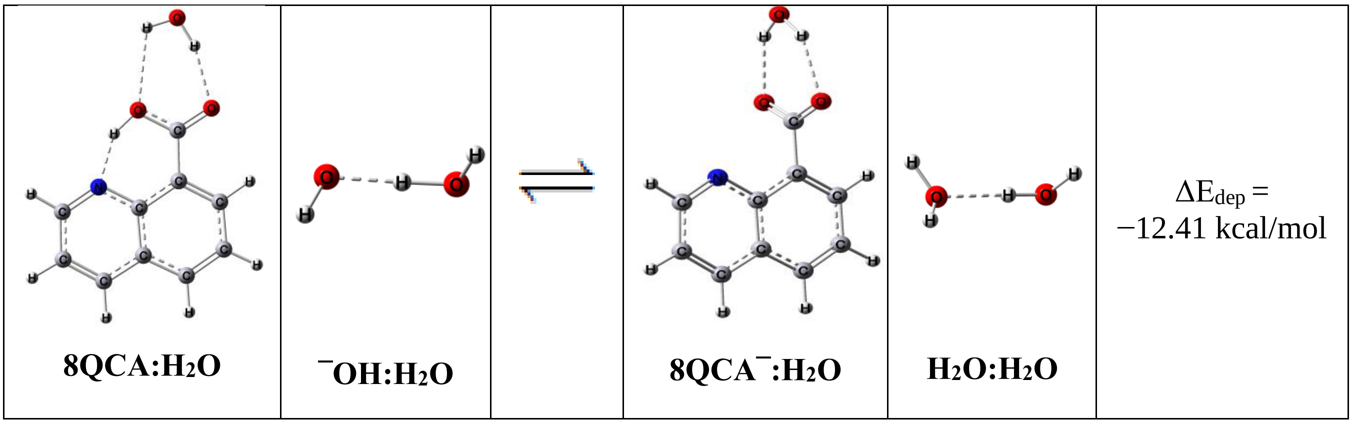

Scheme S2

Table S2: Effective barrier height ($\varepsilon^{\ddagger}$), rate constants using *TST* (k^{TST}) and *d-TST* (k^{d-TST}) methods, diffusion rate constant (k_D) along with the observed rate constants using *TST* (k_{obs}^{TST}) and *d-TST* (k_{obs}^{d-TST}) methods for various carbon atoms in •OH radical addition reaction for neutral 8QCA

	$\varepsilon^{\ddagger}$ (kcal/mol)	k^{TST} ($M^{-1}s^{-1}$)	k^{d-TST} ($M^{-1}s^{-1}$)	k_D ($M^{-1}s^{-1}$)	k_{obs}^{TST} ($1/k^{TST} + 1/k_D$) ($M^{-1}s^{-1}$)	k_{obs}^{d-TST} ($1/k^{d-TST} + 1/k_D$) ($M^{-1}s^{-1}$)
C2	5.56	3.85×10^{5}	4.44×10^{5}	5.76×10^{9}	3.85×10^{5}	4.44×10^{5}
C3	2.52	5.65×10^{7}	6.12×10^{7}	5.89×10^{9}	5.60×10^{7}	6.06×10^{7}
C4	2.86	3.72×10^{7}	3.99×10^{7}	5.75×10^{9}	3.70×10^{7}	3.96×10^{7}
C5	0.73	1.90×10^{9}	1.94×10^{9}	5.77×10^{9}	1.43×10^{9}	1.45×10^{9}
C6	2.37	7.75×10^{7}	8.40×10^{7}	5.87×10^{9}	7.65×10^{7}	8.28×10^{7}
C7	2.51	6.62×10^{7}	7.08×10^{7}	5.86×10^{9}	6.55×10^{7}	7.00×10^{7}
C8	1.20	3.43×10^{8}	3.69×10^{8}	5.76×10^{9}	3.24×10^{8}	3.47×10^{8}
